# Predictors of Delayed Hospital Presentation for Myocardial Infarction Among Diabetic Patients: A Retrospective Study

**DOI:** 10.7759/cureus.112290

**Published:** 2026-07-08

**Authors:** Abdalla A Abdelrahman, Mahmoud R Hamouda

**Affiliations:** 1 Internal Medicine, Al-Azhar University, Cairo, EGY

**Keywords:** delayed, diabetic, infarction, myocardial, patients, predictor, presentation

## Abstract

Background

Myocardial infarction (MI) is a major cause of cardiovascular morbidity and mortality, and early hospital presentation is crucial for timely reperfusion and improved outcomes. Diabetic patients are particularly prone to delayed presentation due to atypical symptoms. Despite this increased risk, limited data are available from developing countries such as Egypt regarding factors associated with delayed hospital presentation. This study aimed to identify predictors of delayed presentation among diabetic patients with MI in Egypt.

Methods

This retrospective study was conducted among 286 diabetic patients with a diagnosis of MI who were admitted to a tertiary care hospital affiliated with Al-Azhar University in Cairo, Egypt. Patients aged ≥30 years with complete records of symptom onset and hospital arrival times were included. MI was diagnosed according to American College of Cardiology/American Heart Association criteria. Patients were classified as early presenters (≤12 hours) or delayed presenters (>12 hours). Sociodemographic and clinical data were collected from medical records. Data were analyzed using the IBM SPSS Statistics for Windows, Version 25 (Released 2017; IBM Corp., Armonk, New York, United States). Chi-square test and multivariable logistic regression analysis were performed, with a p-value ≤0.05 considered statistically significant.

Results

Among 286 diabetic patients with MI, 170 (59.44%) presented late to the hospital, while 116 (40.56%) presented early. On initial analysis, several sociodemographic and clinical factors were significantly associated with delayed presentation, including age ≥60 years, rural residence, low educational level, distance ≥10 km from the hospital, atypical symptom presentation, poor glycemic control (HbA1c ≥7%), and diabetes duration ≥10 years (all p<0.05). On multivariable logistic regression analysis, age ≥60 years (adjusted odds ratio (AOR)=2.43, p=003), rural residence (AOR=1.96, p=0.026), low educational level (AOR=2.18, p=0.010), distance ≥10 km from the hospital (AOR=3.87, p=<0.001), atypical symptoms (AOR=3.15, p=<0.001), HbA1c ≥7% (AOR=2.26, p=0.007), and diabetes duration ≥10 years (AOR=1.84, p=0.039) remained independent predictors of delayed hospital presentation. Conversely, gender, family history of coronary artery disease, hypertension, dyslipidemia, and smoking were not independently associated with delayed presentation of MI.

Conclusion

Delayed hospital presentation among diabetic patients with MI is independently associated with age ≥60 years, rural residence, low educational level, longer distance from the hospital, atypical symptoms, poor glycemic control, and longer diabetes duration. Targeted health education initiatives, improved healthcare accessibility, and enhanced recognition of atypical MI symptoms may help reduce prehospital delays and improve outcomes in this high-risk population.

## Introduction

Myocardial infarction (MI) is a major manifestation of acute coronary syndrome resulting from acute myocardial ischemia due to partial or complete coronary artery occlusion, leading to irreversible myocardial necrosis if not promptly treated. It is classified into ST-segment elevation myocardial infarction (STEMI) and non-ST-segment elevation myocardial infarction (NSTEMI) based on electrocardiographic findings and the extent of coronary obstruction. Both forms are associated with significant morbidity and mortality, with STEMI requiring urgent reperfusion therapy to restore coronary blood flow [1-3].

Cardiovascular disease (CVD) continues to be the foremost cause of illness and death worldwide, responsible for nearly 31% of all global mortality. MI affects an estimated 15.90 million individuals annually and is a major contributor to this burden. The impact is particularly pronounced in low- and middle-income countries [1,4]. In Egypt, CVDs account for 46.20% of all deaths, reflecting a substantial public health challenge further intensified by rising metabolic risk factors, limited preventive care, and delayed access to emergency cardiac services in developing countries [1,5,6].

Risk factors for MI include non-modifiable factors such as age, male sex, and family history, and modifiable factors including diabetes mellitus, hypertension, dyslipidemia, smoking, obesity, and physical inactivity. Among these, diabetes mellitus is particularly important due to its role in accelerating atherosclerosis and its association with silent and atypical myocardial ischemia [7,8]. As a result, diabetic patients frequently present with non-classical symptoms such as dyspnea, fatigue, nausea, dizziness, or epigastric discomfort rather than typical chest pain, contributing to diagnostic delay [9,10].

Delayed presentation in MI is associated with severe complications, including arrhythmias, cardiogenic shock, heart failure, and sudden cardiac death, as well as long-term outcomes such as ventricular remodeling and recurrent ischemia. It also imposes a significant economic burden due to increased emergency care needs, prolonged hospitalization, and intensive care utilization, particularly in resource-limited settings such as Egypt [1,11-16].

Time from symptom onset to hospital presentation is a critical determinant of outcomes, as delays reduce the effectiveness of reperfusion therapy and increase mortality. Previous studies have identified older age, rural residence, low educational level, poor access to healthcare, and greater distance from healthcare facilities as key contributors to delayed presentation. Clinical factors such as poor glycemic control, diabetes mellitus, and comorbid conditions further influence symptom recognition and care-seeking behavior [7-12].

Diabetic patients are especially vulnerable to delay due to a higher prevalence of silent ischemia and atypical symptom presentation [4,11,17]. Compared with non-diabetic individuals, they more frequently present with non-classical symptoms and experience prolonged delays in seeking care [8-10]. Limited health education about identification of symptoms and delayed decision-making, along with system-related barriers such as underutilization of emergency medical services and transport delays, further contribute to prehospital delay [17-20].

Despite global evidence, data from Egypt remain limited, particularly among diabetic patients who represent a high-risk group for delayed and atypical presentation. Therefore, this study aimed to assess sociodemographic and clinical predictors of delayed hospital presentation among diabetic patients with MI in Egypt. The findings may help guide targeted interventions to improve early recognition, reduce prehospital delay, and enhance clinical outcomes in this population.

## Materials and methods

Study design and study population

This retrospective study was conducted at a tertiary care hospital affiliated with the Faculty of Medicine at Al-Azhar University, Cairo, Egypt, over a period of 24 months from July 2022 to July 2024. Out of 356 patients screened for eligibility, 286 diabetic patients with a confirmed diagnosis of MI who fulfilled the predefined inclusion and exclusion criteria were enrolled consecutively in the study. Institutional ethical clearance and patient informed consent were secured prior to study initiation.

Inclusion and exclusion criteria

**Figure 1:**
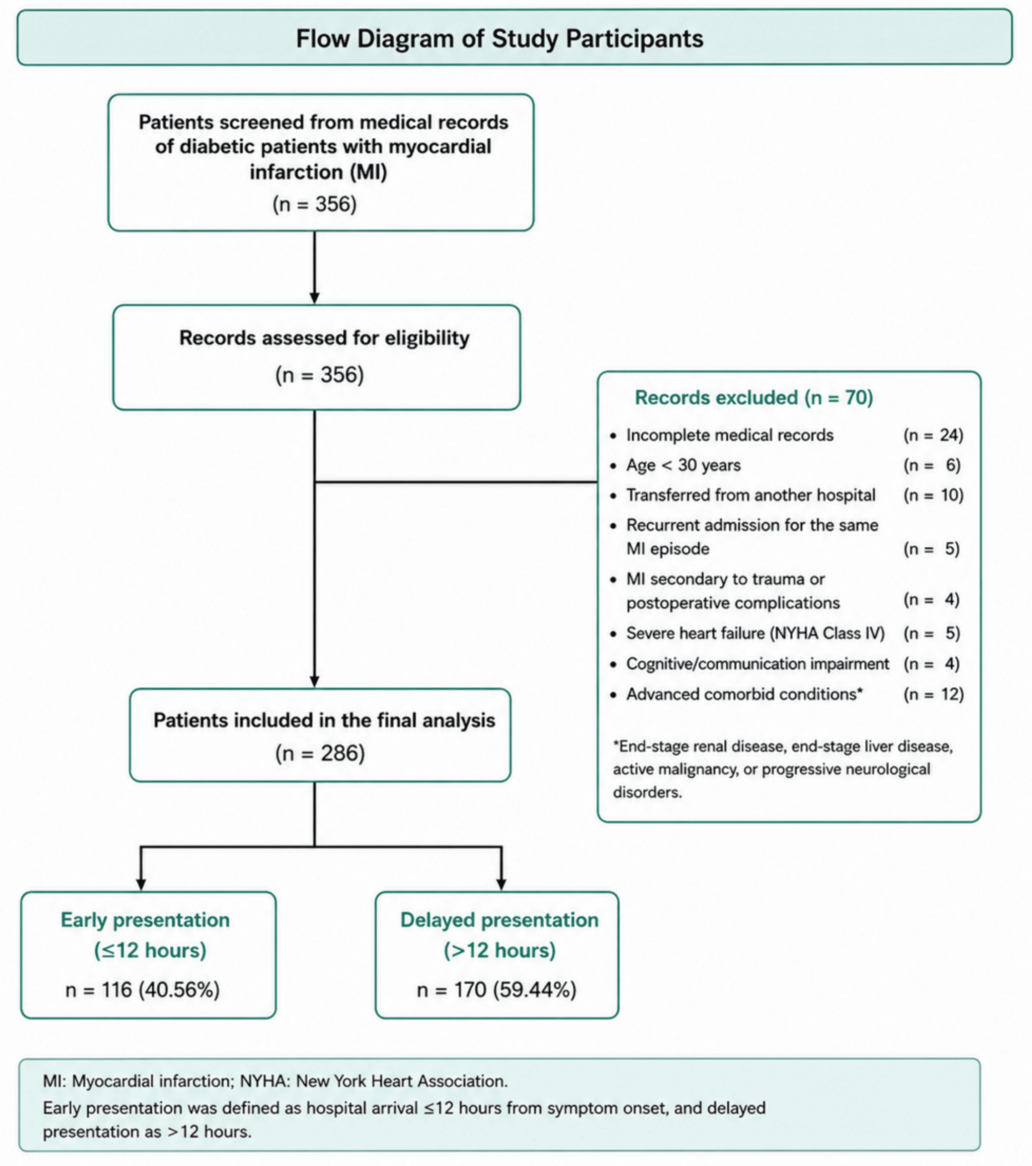
Participant Flow Diagram Illustrating Patient Screening, Eligibility Assessment, Exclusions, and Final Inclusion in the Study

Diabetic patients aged ≥30 years with a confirmed diagnosis of MI and with complete records, including documented symptom onset and hospital arrival times, were included. Patients were excluded if they were transferred from another healthcare facility, had recurrent admissions for the same MI episode, had MI secondary to trauma or postoperative complications, had severe heart failure as per New York Heart Association classification (NYHA) Class IV [21,22], cognitive or communication impairment affecting history reliability, or had advanced comorbid conditions including end-stage renal disease, end-stage liver disease, active malignancy, or progressive neurological disorders, to minimize potential confounding factors. In Figure 1, a study flow diagram shows the inclusion and exclusion criteria section to improve the transparency of participant selection and study reporting.

Primary and secondary outcomes

The primary outcome of the study was the frequency of delayed hospital presentation in diabetic patients with MI. The secondary outcome aimed to identify the risk factors associated with delayed presentation of MI among diabetic patients.

Data collection

MI was diagnosed according to the Fourth Universal Definition of Myocardial Infarction, based on the presence of clinical symptoms, electrocardiographic changes, and elevated cardiac biomarkers [23,24]. In this study, early presentation was defined as arrival at the emergency department within 12 hours of symptom onset, whereas delayed presentation was defined as arrival more than 12 hours after symptom onset. Although the greatest benefit of reperfusion therapy is achieved within the first few hours after symptom onset, the 12-hour threshold has been widely used in previous studies evaluating delayed hospital presentation, facilitating comparison across studies [4,17]. Data collected from institutional medical records using a self-structured data collection proforma. The proforma comprised two sections: the initial noted history; the latter included findings from diagnostic investigations. The demographic variables included age (<60 years and ≥60 years), gender (male and female), education level (illiterate or middle (low level) and matriculation or above (high level)), residence (rural and urban), and distance from the hospital (<10 km and ≥10 km). Clinical characteristics included the type of MI symptoms (patients were categorized as having typical symptoms if they presented with chest pain or discomfort (central or left-sided, with or without radiation to the left arm, shoulder, neck, jaw, or back) while atypical symptoms were defined as presentations without typical ischemic chest pain, including dyspnea, epigastric discomfort, nausea or vomiting, unexplained fatigue, dizziness or syncope, and diaphoresis without chest pain) [7,9], glycated hemoglobin levels (HbA1c <7% and ≥7%), duration of diabetes mellitus (<10 years and ≥10 years), and the presence of comorbid conditions such as family history of coronary artery disease, dyslipidemia, hypertension, and smoking status (all categorized as yes or no).

Data analysis

Data analysis was conducted using IBM SPSS Statistics for Windows, Version 25 (Released 2017; IBM Corp., Armonk, New York, United States). The normality of variables was assessed using the Shapiro-Wilk test. Continuous data were presented as mean ± standard deviation, while categorical variables were expressed as frequencies and percentages. For group comparisons, the chi-square test was applied to compare study variables between the study groups. Variables selected based on clinical relevance and statistical significance in the univariable analysis (p≤0.05) were simultaneously entered into the multivariable logistic regression model using the enter method to identify independent predictors of delayed hospital presentation of MI among diabetic patients. A p-value of ≤0.05 was considered statistically significant.

## Results

A total of 286 diabetic patients with MI were included, of whom 170 (59.44%) had delayed hospital presentation (>12 hours) and 116 (40.56%) presented within 12 hours. The mean ± SD values for age, duration of diabetes, HbA1c, and distance from hospital were 61.78 ± 8.89 years, 12.24 ± 5.14 years, 8.40 ± 1.15%, and 18.55 ± 12.61 km, respectively.

Table 1 shows that delayed presentation of MI was significantly associated with age ≥60 years, rural residence, lower educational attainment, and living ≥10 km from the hospital, while no significant association was observed with gender.

**Table 1:** Role of Sociodemographic Characteristics in the Presentation Time of Myocardial Infarction Patients

Variable	Category	Early Presentation (n=116)	Delayed Presentation (n=170)	Test Statistic (χ^2^)	p-value
Age	<60 years	74 (63.80%)	62 (36.47%)	20.64	<0.001
	≥60 years	42 (36.20%)	108 (63.53%)		
Gender	Female	37 (31.90%)	50 (29.42%)	0.20	0.638
	Male	79 (68.10%)	120 (70.58%)		
Residence	Urban	69 (59.48%)	70 (41.18%)	9.25	0.002
	Rural	47 (40.52%)	100 (58.82%)		
Education	Higher education	61 (52.58%)	46 (27.06%)	19.20	<0.001
	Low education	55 (47.42%)	124 (72.94%)		
Distance from hospital	<10 km	73 (62.93%)	51 (30.0%)	30.45	<0.001
	≥10 km	43 (37.07%)	119 (70.0%)		

Table 2 demonstrates that delayed presentation was significantly associated with atypical symptom presentation, poor glycemic control, and diabetes duration ≥10 years. In contrast, family history of coronary artery disease, hypertension, dyslipidemia, and smoking status showed no significant differences between the groups.

**Table 2:** Role of Clinical Characteristics in the Presentation Time of Myocardial Infarction Patients CAD: coronary artery disease

Variable	Category	Early Presentation (n=116)	Delayed Presentation (n=170)	Test Statistic (χ^2^)	p-value
Symptoms	Typical	89 (76.72%)	76 (44.70%)	29.27	<0.001
	Atypical	27 (23.28%)	94 (55.30%)		
HbA1c	<7%	55 (47.41%)	37 (21.76%)	21.45	<0.001
	≥7%	61 (52.59%)	133 (78.24%)		
Diabetes duration	<10 years	77 (66.37%)	79 (46.47%)	11.32	0.001
	≥10 years	39 (33.63%)	91 (53.53%)		
Family history of CAD	No	92 (79.31%)	131 (77.05%)	0.23	0.633
	Yes	24 (20.69%)	39 (22.95%)		
Hypertension	No	49 (42.24%)	62 (36.48%)	1.06	0.304
	Yes	67 (57.76%)	108 (63.52%)		
Dyslipidemia	No	54 (46.55%)	66 (38.82%)	1.86	0.172
	Yes	62 (53.45%)	104 (61.18%)		
Smoking	No	66 (56.89%)	88 (51.76%)	0.80	0.372
	Yes	50 (43.11%)	82 (48.24%)		

Table 3 indicates that age ≥60 years, rural residence, low educational level, distance ≥10 km from the hospital, atypical symptoms, HbA1c ≥7%, and diabetes duration ≥10 years were independent predictors of delayed presentation. Among these, distance ≥10 km and atypical symptom presentation showed the strongest associations with delayed hospital presentation, whereas male gender, hypertension, dyslipidemia, smoking, and family history of coronary artery disease were not independently associated with delay.

**Table 3:** Multivariable Logistic Regression Analysis of Predictors Associated With Delayed Hospital Presentation of Myocardial Infarction Among Diabetic Patients CAD: coronary artery disease; OR: odds ratio; HbA1c: glycated hemoglobin; CI: confidence interval

Variable	Crude OR	Crude 95% CI	Crude p-value	Adjusted OR	Adjusted 95% CI	Adjusted p-value
Age ≥60 years	3.06	1.85-5.06	<0.001	2.43	1.34-4.42	0.003
Male gender	1.13	0.68-1.88	0.638	1.19	0.68-2.08	0.541
Rural residence	2.09	1.30-3.36	0.002	1.96	1.08-3.56	0.026
Low education	2.99	1.85-4.83	<0.001	2.18	1.21-3.94	0.010
Distance ≥10 km	3.95	2.44-6.40	<0.001	3.87	2.02-7.42	<0.001
Atypical symptoms	4.03	2.45-6.63	<0.001	3.15	1.71-5.80	<0.001
HbA1c ≥7%	3.23	1.98-5.28	<0.001	2.26	1.24-4.10	0.007
Diabetes duration ≥10 years	2.27	1.40-3.68	0.001	1.84	1.03-3.29	0.039
Family history of CAD	1.14	0.66-1.98	0.633	1.22	0.64-2.31	0.544
Hypertension	1.27	0.80-2.03	0.304	1.14	0.65-2.01	0.647
Dyslipidemia	1.37	0.87-2.17	0.172	1.08	0.61-1.91	0.788
Smoking	1.23	0.78-1.94	0.372	1.29	0.73-2.29	0.381

## Discussion

This present study evaluated predictors of delayed hospital presentation among diabetic patients with MI and demonstrated that delayed presentation is highly prevalent and driven by a combination of sociodemographic, geographic, clinical, and healthcare access-related factors. The strongest independent predictors of delay were distance from hospital and atypical symptom presentation, followed by older age, rural residence, low educational level, poor glycemic control, and longer duration of diabetes. These findings highlight the multifactorial nature of prehospital delay in a high-risk diabetic population and reflect both patient-related and system-related barriers [7-10].

More than half of the patients (59.44%) presented after 12 hours of symptom onset. A similar frequency of delayed presentation among patients with MI has been reported in a previous study [4]. Although lower rates have also been observed elsewhere [17], such variation may reflect differences in sociodemographic characteristics, healthcare accessibility, emergency medical services, and public awareness across regions [18,19].

Age ≥60 years was significantly associated with delayed presentation. Older individuals often have reduced symptom perception, atypical disease manifestations, cognitive delays in decision-making, and a higher burden of comorbidities, all of which may contribute to delayed care-seeking. Similar associations have been reported in studies from different populations, where increasing age was linked to prolonged prehospital delay due to misinterpretation of ischemic symptoms and limited education about the identification of symptoms [4,11]. Although MI was more common among males, gender was not significantly associated with delayed hospital presentation in the present study, a finding that is consistent with previous reports [9,12].

Rural residence, low educational level, and greater distance from healthcare facilities were also strongly associated with delayed presentation. These findings reflect structural barriers to emergency cardiac care and have been consistently reported in low- and middle-income countries, where limited health literacy of MI symptoms, transportation challenges, and reduced healthcare utilization contribute to delayed presentation [13,17]. Geographic distance remains one of the most consistent predictors of delay in acute coronary syndrome populations, emphasizing the need for efficient transport systems and regional cardiac care networks [12,20].

Among clinical factors, atypical symptom presentation was a major determinant of delay. Diabetic patients frequently experience silent ischemia and non-classical symptoms because of autonomic neuropathy, leading to under-recognition of MI and delayed hospital arrival. Similar findings have been reported in previous studies demonstrating higher rates of atypical presentations and prolonged delay among diabetic patients [7,9,10,12]. Poor glycemic control and longer duration of diabetes were also significantly associated with delayed presentation, likely reflecting advanced disease, microvascular complications, and impaired pain perception, findings that are consistent with earlier reports [8,11].

Interestingly, traditional cardiovascular risk factors, including family history of coronary artery disease, hypertension, dyslipidemia, and smoking, were not independently associated with delayed presentation. This suggests that healthcare access, symptom recognition, and educational factors may exert a greater influence on healthcare-seeking behavior than conventional cardiovascular risk factors, a finding also reported in previous studies [4,13,17].

The observed delays likely result from a combination of patient- and system-level factors. Poor recognition of atypical symptoms, geographic barriers, transportation limitations, underutilization of emergency medical services, and diabetic autonomic neuropathy may all contribute to delayed care-seeking and hospital arrival [7-11,21].

The findings have important clinical and public health implications. Most identified predictors can be readily assessed during routine clinical encounters, allowing early identification of individuals at high risk of delayed presentation. Targeted educational programs, particularly for elderly, rural, and poorly educated diabetic patients, together with improvements in emergency transport services and regional cardiac care networks, may help reduce prehospital delays.

This study's several limitations should be considered when interpreting the findings. The retrospective single-center design may limit the generalizability of the results. In addition, selection bias may have occurred because only eligible patients with complete medical records who reached the hospital were included, potentially introducing survivorship bias by excluding patients who died before hospital arrival. Unmeasured factors, including health literacy, psychosocial influences, cultural beliefs, and emergency medical service utilization, may have contributed to residual confounding despite multivariable adjustment. Furthermore, reliance on medical records limited the detailed assessment of the decision-making process between symptom onset and hospital presentation.

Despite these limitations, this study provides valuable evidence regarding the determinants of delayed hospital presentation among diabetic patients with MI. The findings support a comprehensive approach that combines patient education, early risk identification, and healthcare system improvements. Future prospective multicenter studies are warranted to validate these findings and evaluate interventions aimed at reducing prehospital delay and improving cardiovascular outcomes in this high-risk population.

## Conclusions

This study shows that delayed hospital presentation among diabetic patients with MI was highly prevalent and was independently associated with older age, rural residence, lower educational attainment, greater distance from the hospital, poor glycemic control, longer duration of diabetes, and atypical symptom presentation. Distance from the hospital and atypical symptoms were the strongest predictors of delay. Importantly, most of the identified predictors can be readily recognized through routine clinical evaluation and history-taking, making early risk stratification practical even in resource-constrained healthcare settings. Prompt identification of high-risk individuals, particularly diabetic patients with atypical symptom presentations, together with improved public education of MI symptoms, may facilitate earlier healthcare-seeking behavior and timely access to reperfusion therapy.

These findings underscore the need for targeted interventions to enhance patient education, strengthen primary healthcare referral systems, and improve access to emergency cardiac services, particularly in rural and underserved regions. Such measures have the potential to reduce prehospital delays and improve clinical outcomes among diabetic patients with acute MI.
